# Anti-tumor activities of the new oral pan-RAF inhibitor, TAK-580, used as monotherapy or in combination with novel agents in multiple myeloma

**DOI:** 10.18632/oncotarget.27775

**Published:** 2020-11-03

**Authors:** Rikio Suzuki, Yuka Kitamura, Yoshihiko Nakamura, Hibiki Akashi, Yoshiaki Ogawa, Hiroshi Kawada, Kiyoshi Ando

**Affiliations:** ^1^Department of Hematology/Oncology, Tokai University School of Medicine, Isehara, Kanagawa, Japan; ^2^Center for Regenerative Medicine, Tokai University School of Medicine, Isehara, Kanagawa, Japan

**Keywords:** pan-RAF inhibitor, Bim, FOXO3a, bortezomib, lenalidomide

## Abstract

Many RAS pathway inhibitors, including pan-RAF inhibitors, have shown significant anti-tumor activities in both solid and hematological tumors. The pan-RAF inhibitor, TAK-580, is a representative of the novel RAF inhibitors that act by disrupting RAF homo- or heterodimerization. In this study, we examined the anti-tumor effects of TAK-580 used as monotherapy or in combination with bortezomib, lenalidomide, or other novel agents in multiple myeloma (MM) cells *in vitro*. TAK-580 monotherapy potently targeted proteins in the RAS-RAF-MEK-ERK signaling pathway and induced potent cytotoxicity and apoptosis in MM cell lines and myeloma cells from patients with newly diagnosed and relapsed and/or refractory MM, compared with a representative RAF inhibitor, dabrafenib. Normal donor peripheral blood B lymphocytes and cord blood CD34-positive cells were not affected. Importantly, TAK-580 significantly inhibited phospho-FOXO3 and induced upregulation of Bim_L_ and Bim_S_ in a dose-dependent manner, finally leading to apoptosis in MM cells. Moreover, TAK-580 enhanced bortezomib-induced cytotoxicity and apoptosis in MM cells via the FOXO3-Bim axis and the terminal unfolded protein response. Importantly, TAK-580 also enhanced lenalidomide-induced cytotoxicity and apoptosis in MM cells. Taken together, our results provide the rationale for TAK-580 monotherapy and/or treatment in combination with novel agents to improve outcomes in patients with MM.

## INTRODUCTION

Multiple myeloma (MM) is a B-cell malignancy characterized by clonal proliferation of abnormal plasma cells driven by genetic abnormalities in the specific bone marrow microenvironment and niche, excess production of monoclonal protein in the serum and/or urine, osteolytic bone lesions, and immunodeficiency [1, 2]. In recent years, a revolution in treatment strategies has prolonged the survival of patients with both newly diagnosed and relapsed/refractory MM. These treatments include high-dose chemotherapy and stem cell transplantation; immunotherapies including two monoclonal antibodies (daratumumab and elotuzumab), bi-specific T-cell engagers, and chimeric antigen receptor therapy; and novel therapies including bortezomib (BTZ), thalidomide, and lenalidomide (LEN) [1, 3, 4]. However, MM is still considered an incurable disease, and the advent of next-generation novel therapies is awaited.

The rat sarcoma (RAS)-v-raf murine sarcoma viral oncogene homolog (RAF)-mitogen-activated protein kinase/extracellular signal-regulated kinase kinase (MEK)-extracellular signal-regulated kinase (ERK) signaling pathway is one of the most extensively characterized pathways in oncogenesis and plays a crucial role in the regulation of cell proliferation, survival, and motility/invasion [5]. The RAS pathway plays a main role in the transformation from monoclonal gammopathy of undetermined significance (MGUS) to MM. Activating *RAS* mutations (mainly *neuroblastoma ras viral oncogene homolog* (*NRAS*) or *v-ki-ras2 kirsten rat sarcoma viral oncogene homolog* (*KRAS*)) are detected in 32–50% of patients with MM [6]. Furthermore, >70% of patients have *RAS*/*RAF* mutations present at relapse [7]. Patients with *RAS* mutations have significantly shorter overall survival (OS) and progression-free survival, indicating that *RAS* mutations are an independent prognostic factor in MM [8], and *NRAS* mutations significantly decrease the sensitivity of myeloma to single-agent BTZ therapy [9]. Therefore, the RAS-RAF-MEK-ERK pathway is believed to be a promising target for anti-MM therapy [7]. Many RAS pathway inhibitors, including RAF inhibitors and MEK inhibitors, have been developed [5]. However, RAS pathway-driven cancers, including MM, are particularly aggressive and are refractory to current therapeutic interventions (mainly RAF and MEK inhibitors), typically due to narrow therapeutic windows, paradoxical pathway activation, feedback induction of phosphatidylinositol-3 kinase/Akt signaling, and/or drug resistance, demonstrating an unmet medical need [10]. Therefore, the best way to use RAF/MEK inhibitors to overcome these resistance mechanisms has yet to be elucidated. Importantly, TAK-580 (Supplementary Figure 1), which is a representative of a novel category of RAF inhibitors, acts by disrupting RAF homo- or heterodimerization and leads to inhibition of MEK, because TAK-580 can interfere with signaling through wild-type RAF as well as mutant RAF [11]. Moreover, TAK-580 efficiently crosses the blood-brain barrier [12, 13] and significantly inhibits pediatric low-grade astrocytoma cells with BRAFV600E *in vitro* and *in vivo* [12]. Therefore, TAK-580 is expected to be a promising pan-RAF inhibitor.

In the present study, we demonstrate that TAK-580, alone or in combination with novel agents, shows significant synergistic anti-myeloma effects in MM cells *in vitro*, providing the framework for its clinical evaluation to improve MM patient outcome.

## RESULTS

### TAK-580 specifically inhibits the RAS-RAF-MEK-ERK pathway and induces anti-myeloma effects in MM cells

**Figure 1:**
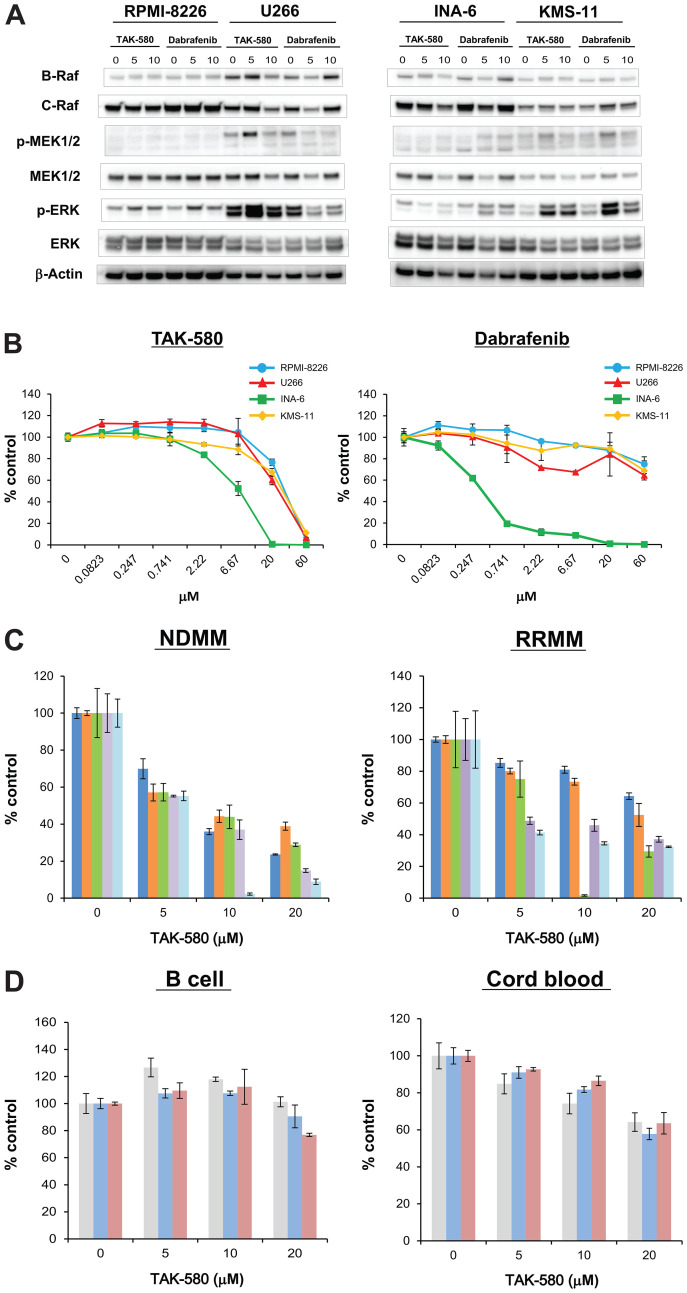
TAK-580 specifically inhibits the RAS-RAF-MEK-ERK pathway and induces anti-myeloma effects in MM cells. (**A**) RPMI-8226, U266, INA-6, and KMS-11 MM cell lines were treated with the indicated doses of TAK-580 (0–10 μM) or dabrafenib (0–10 μM) for 24 h. Whole-cell lysates were subjected to western blotting using B-Raf, C-Raf, p-MEK1/2, MEK1/2, p-ERK, ERK, and β-Actin Abs. (**B**) RPMI-8226, U266, INA-6, and KMS-11 MM cell lines were cultured with TAK-580 (0–60 μM) or dabrafenib (0–60 μM) for 48 h. In each case, the cell viability of triplicate cultures was assessed with the Cell Titer-Glo^®^ Cell Viability Assay and expressed as the percentage of the untreated control. Data are the mean ± standard deviation (SD). (**C**) Cells from five patients with newly diagnosed multiple myeloma (NDMM) and five patients with relapsed/refractory multiple myeloma (RRMM) were cultured with TAK-580 (0–20 μM) for 48 h. In each case, the cell viability of triplicate cultures was assessed with the Cell Titer-Glo^®^ Cell Viability Assay and expressed as the percentage of the untreated control. Data are the mean ± SD. (**D**) Peripheral blood B lymphocytes from three normal donors and CD34-positive cells from three donors of cord blood were cultured with TAK-580 (0–20 μM) for 48 h. In each case, the cell viability of triplicate cultures was assessed with the Cell Titer-Glo^®^ Cell Viability Assay and expressed as the percentage of the untreated control. Data are the mean ± SD.

First, we assessed whether the pan-RAF inhibitor, TAK-580, specifically inhibits key proteins in the RAS-RAF-MEK-ERK pathway in MM cells using western blot analysis. Significant degradation of key RAS-RAF-MEK-ERK pathway regulators (B-Raf, C-Raf, phospho-MEK, and phospho-ERK) was triggered by TAK-580 in a dose-dependent manner in MM cell lines, especially in U266 and INA-6, compared with dabrafenib, a representative RAF inhibitor (Figure 1A).

We next examined the growth inhibitory effect of TAK-580 in MM cells. TAK-580 significantly inhibited growth of these MM cell lines in a dose-dependent manner compared with dabrafenib (Figure 1B). In addition, TAK-580 also inhibited growth of KMS-11 and U266 cells in a time-dependent manner (Supplementary Figure 2). Moreover, TAK-580 also potently inhibited newly diagnosed MM and relapsed/refractory MM (RRMM) cells from patients (Figure 1C), without affecting normal donor peripheral blood B lymphocytes and cord blood CD34-positive cells (Figure 1D). Taken together, these results indicate that TAK-580 potently targets RAS-RAF-MEK-ERK signaling pathway proteins and induces potent cytotoxicity in MM cells.

### TAK-580 induces apoptosis in MM cells

**Figure 2:**
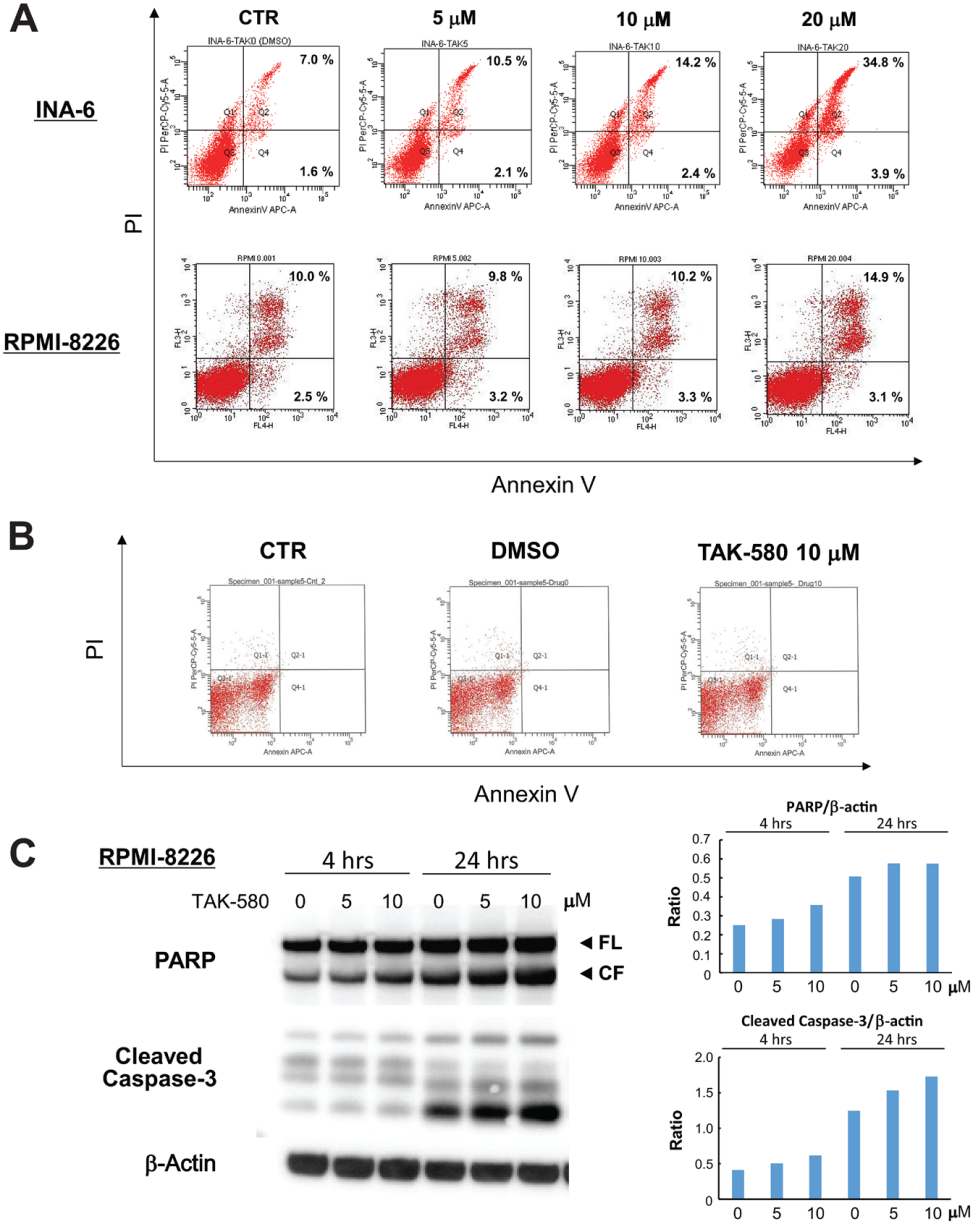
TAK-580 induces apoptosis in MM cells. (**A**) INA-6 cells were treated with TAK-580 (0–20 μM) for 24 h; RPMI-8226 cells were treated with TAK-580 (0–20 μM) for 48 h. Apoptotic cells were analyzed with flow cytometry using annexin V/PI staining. Apoptosis was assessed as the percentage of annexin V-positive cells. (**B**) Normal donor peripheral blood B lymphocytes were treated with dimethylsulfoxide or TAK-580 (10 μM) for 48 h. Apoptotic cells were analyzed with flow cytometry using annexin V/PI staining. Apoptosis was assessed as the percentage of annexin V-positive cells. (**C**) RPMI-8226 cells were treated with TAK-580 (0–10 μM) for 4 or 24 h. Whole-cell lysates were subjected to western blotting using PARP, cleaved caspase-3, and β-Actin Abs. FL, full-length; CF, cleaved form. (Upper right panel): The graph represents ratios of PARP CF density relative to β-Actin in Figure 2C. (Lower right panel): The graph represents ratios of cleaved caspase-3 density relative to β-Actin in Figure 2C.

We next investigated the mechanism of cytotoxicity triggered by TAK-580 using annexin V/PI staining and western blotting in MM cells. The analysis showed a significant dose-dependent increase in annexin V-positive cells after treatment with TAK-580 in INA-6 and RPMI-8226 cells (Figure 2A), without affecting normal donor peripheral blood B lymphocytes (Figure 2B). In addition, TAK-580 markedly induced caspase-3 and PARP cleavage in RPMI-8226 cells in a dose- and time-dependent manner (Figure 2C). Taken together, these results strongly suggest that TAK-580 triggers caspase-dependent apoptosis in MM cells.

### TAK-580 induces apoptosis via the FOXO3-Bim axis in MM cells

**Figure 3:**
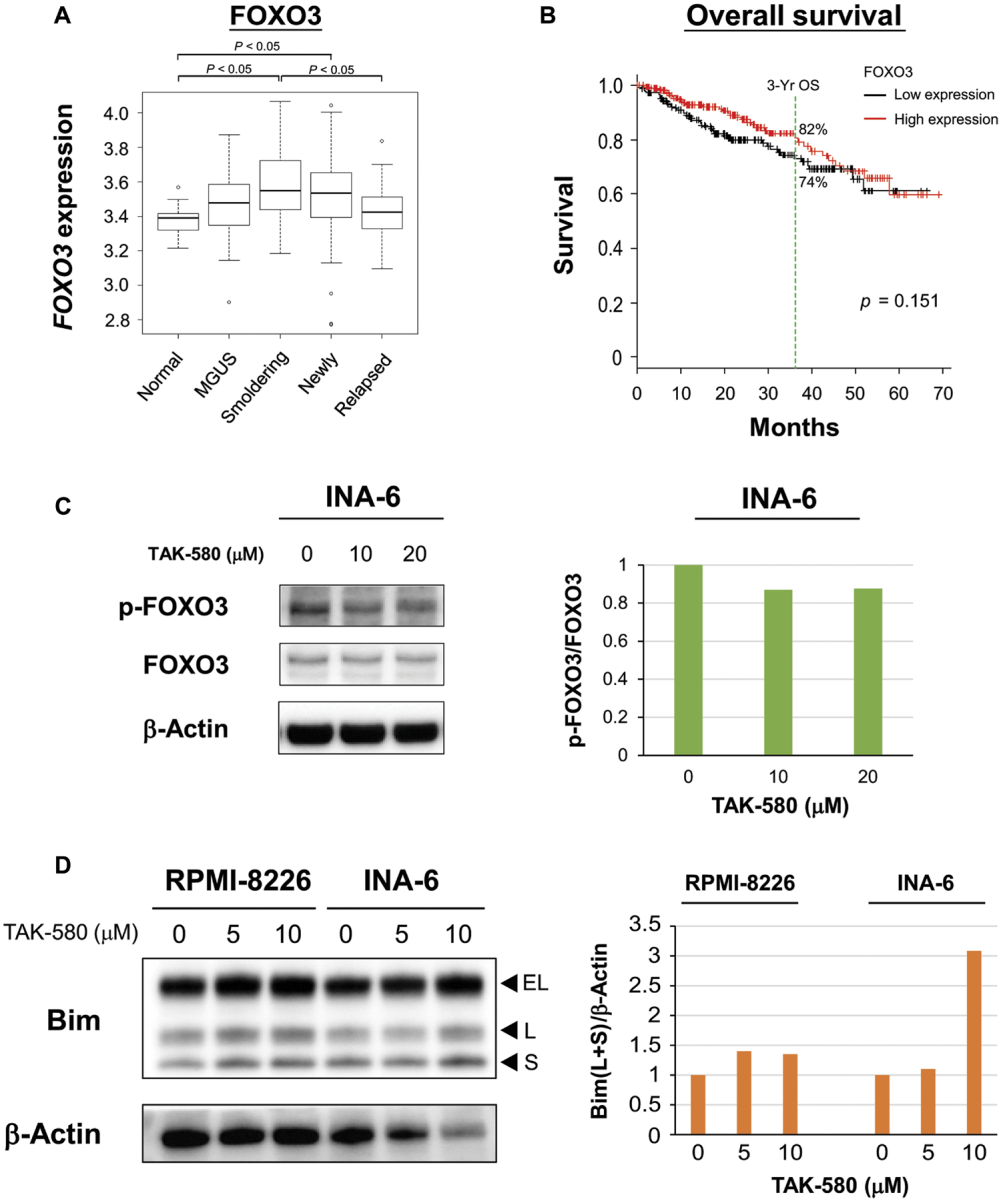
TAK-580 induces apoptosis via the FOXO3-Bim axis in MM cells. (**A**) Publicly available microarray GSE6477 data sets were analyzed for mRNA expression of *FOXO3* in normal plasma cells (Normal), monoclonal gammopathy of undetermined significance (MGUS), smoldering multiple myeloma (Smoldering), newly diagnosed multiple myeloma (Newly), and relapsed multiple myeloma (Relapsed) using the Kruskal-Wallis test. (**B**) Kaplan-Meier overall survival curves of MM patients according to *FOXO3* expression above or below the value of 4000, based on gene expression omnibus dataset GSE4581. The black line indicates the patient group with lower *FOXO3* expression, whereas the red line represents the group of patients with higher *FOXO3* expression. (**C**) (Left panel): INA-6 cells were treated with TAK-580 (0–20 μM) for 3 h. Whole-cell lysates were subjected to western blotting using phospho-FOXO3 (p-FOXO3), FOXO3, and β-Actin Abs. (Right panel): The graph represents ratios of p-FOXO3 density relative to FOXO3 in Figure 3C. (**D**) (Left panel): RPMI-8226 and INA-6 cells were treated with TAK-580 (0–10 μM) for 48 h. Whole-cell lysates were subjected to western blotting using Bim and β-Actin Abs. Bim has three main isoforms generated by alternative splicing: Bim_EL_, Bim_L_, and the most pro-apoptotic variant, Bim_S_. (Right panel): The graph represents ratios of Bim_(L+S)_ density relative to β-Actin in Figure 3D.

We next examined the mechanism of apoptosis induced by TAK-580 in MM cell lines. We evaluated expression of *FOXO3* mRNA in MM patient samples using publicly available gene expression profiling data because FOXO3 has been implicated in the pathogenesis of several other cancers [14]. In the GSE6477 data set, *FOXO3* expression was significantly elevated in smoldering and newly diagnosed MM patient samples compared with normal plasma cells (Figure 3A). On the other hand, *FOXO3* expression was significantly decreased in relapsed MM patient samples compared with smoldering MM patient samples (Figure 3A). Moreover, when we subdivided MM samples into two groups based on *FOXO3* expression, individuals with low expression (3-year OS 74.4%, 95% confidence interval: 66.1–81.0) tended to have shorter survival than those with high expression (3-year OS 82.4%, 95% confidence interval: 74.7–88.0) (Figure 3B). These results indicated that FOXO3 is involved in early stage disease progression of MM; however, a decrease in FOXO3 may be a poor prognostic factor for OS when the degree of dependence on the FOXO3 pathway decreases.

Wang et al. reported that FOXO3-mediated upregulation of Bim is a key mechanism for cancer cell apoptosis [15]. Therefore, we next examined the effect of TAK-580 on the FOXO3-Bim axis in MM cells. Interestingly, TAK-580 inhibited phospho-FOXO3 in a dose-dependent manner in INA-6 cells (Figure 3C). Bim has three main isoforms generated by alternative splicing: Bim_EL_, Bim_L_, and the most pro-apoptotic variant, Bim_S_ [16, 17]. We also confirmed that TAK-580 significantly induced upregulation of Bim_L_ and Bim_S_ in a dose-dependent manner in RPMI-8226 and INA-6 cells (Figure 3D). Taken together, these results indicate that TAK-580 triggers cytotoxicity and induces apoptosis via the FOXO3-Bim axis in MM cells, especially in the early stage of pathogenesis of MM.

### The combination of TAK-580 and BTZ triggers synergistic anti-MM activity

**Figure 4:**
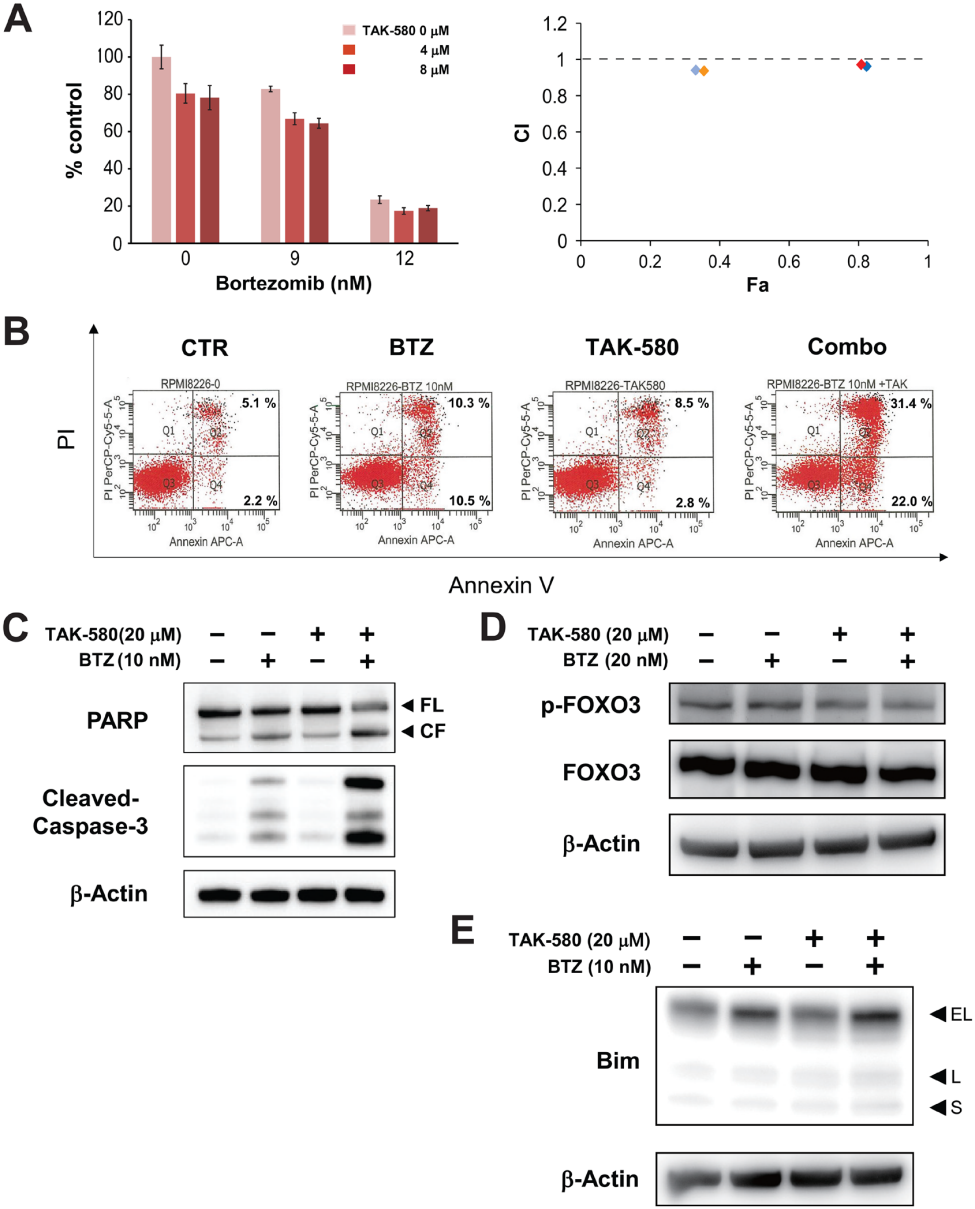
The combination of TAK-580 and BTZ triggers synergistic anti-MM activity. (**A**) (Left panel): KMS-11 cells were treated with TAK-580 (0–8 μM) in combination with BTZ (0–12 nM) for 48 h. In each case, the cell viability of triplicate cultures was assessed with the Cell Titer-Glo^®^ Cell Viability Assay and expressed as the percentage of the untreated control. Data are the mean ± SD. (Right panel): Isobologram analysis shows the synergistic or additive cytotoxic effect of TAK-580 and BTZ. Light blue, blue, orange, and red rhombuses indicate CI values of the combination of TAK-580 and BTZ; 0.94 (TAK-580 4 μM and BTZ 9 nM), 0.96 (TAK-580 4 μM and BTZ 12 nM), 0.94 (TAK-580 8 μM and BTZ 9 nM), and 0.97 (TAK-580 4 μM and BTZ 12 nM), respectively. CI < 1.0 indicates synergism; CI = 1.0 indicates an additive effect; and CI > 1.0 indicates antagonism. CI, combination index; Fa, fraction affected. (**B**) RPMI-8226 cells were cultured with TAK-580 (20 μM), BTZ (10 nM), or TAK-580 plus BTZ for 24 h. Apoptotic cells were analyzed with flow cytometry using annexin V/PI staining. Apoptosis was assessed as the percentage of annexin V-positive cells. (**C**) RPMI-8226 cells were treated with TAK-580 (20 μM) alone or in combination with BTZ (10 nM) for 24 h. Whole-cell lysates were subjected to western blotting using PARP, cleaved caspase-3, and β-Actin Abs. FL, full-length; CF, cleaved form. (**D**) KMS-11 cells were treated with TAK-580 (20 μM) alone or in combination with BTZ (20 nM) for 5 h. Whole-cell lysates were subjected to western blotting using phospho-FOXO3, FOXO3, and β-Actin Abs. (**E**) RPMI-8226 cells were treated with TAK-580 (20 μM) alone or in combination with BTZ (10 nM) for 5 h. Whole-cell lysates were subjected to western blotting using Bim and β-Actin Abs. Bim has three main isoforms generated by alternative splicing: Bim_EL_, Bim_L_, and the most pro-apoptotic variant, Bim_S_.

We next assessed the anti-MM effect of TAK-580 in combination with BTZ using the Cell Titer-Glo^®^ Cell Viability assay. The combination of TAK-580 plus BTZ induced additive or synergistic cytotoxicity in KMS-11 cells (Figure 4A). In addition, annexin V/PI staining showed that TAK-580 significantly enhanced apoptosis induced by BTZ in RPMI-8226 cells (Figure 4B).

We then examined the combination effect on the modulation of the FOXO3-Bim axis in MM cell lines using western blot analysis. Importantly, cleavage of PARP and caspase-3 was significantly enhanced by TAK-580 in combination with BTZ in RPMI-8226 cells (Figure 4C). In addition, TAK-580 and BTZ synergistically inhibited phospho-FOXO3 in KMS-11 cells (Figure 4D; Supplementary Figure 3A). Furthermore, Bim expression was enhanced by TAK-580 in combination with BTZ in RPMI-8226 cells (Figure 4E). We also examined the combination effect on endoplasmic reticulum (ER) stress, because the unfolded protein response (UPR) induced by excessive ER stress is one of the major apoptosis mechanisms for BTZ [18]. Importantly, CHOP, a major ER stress-mediated apoptosis executer, was significantly enhanced by TAK-580 in combination with BTZ in RPMI-8226 cells (Supplementary Figure 3C). On the other hand, although TAK-580 or BTZ alone induced p-AKT via feedback activation, p-AKT was not enhanced by this combination in KMS-11 cells (Supplementary Figure 3B). Taken together, these results indicated that TAK-580 enhanced BTZ-induced cytotoxicity and apoptosis in MM cells via the FOXO3-Bim axis and the terminal UPR.

### TAK-580 induces synergistic cytotoxicity with LEN in MM cells

**Figure 5:**
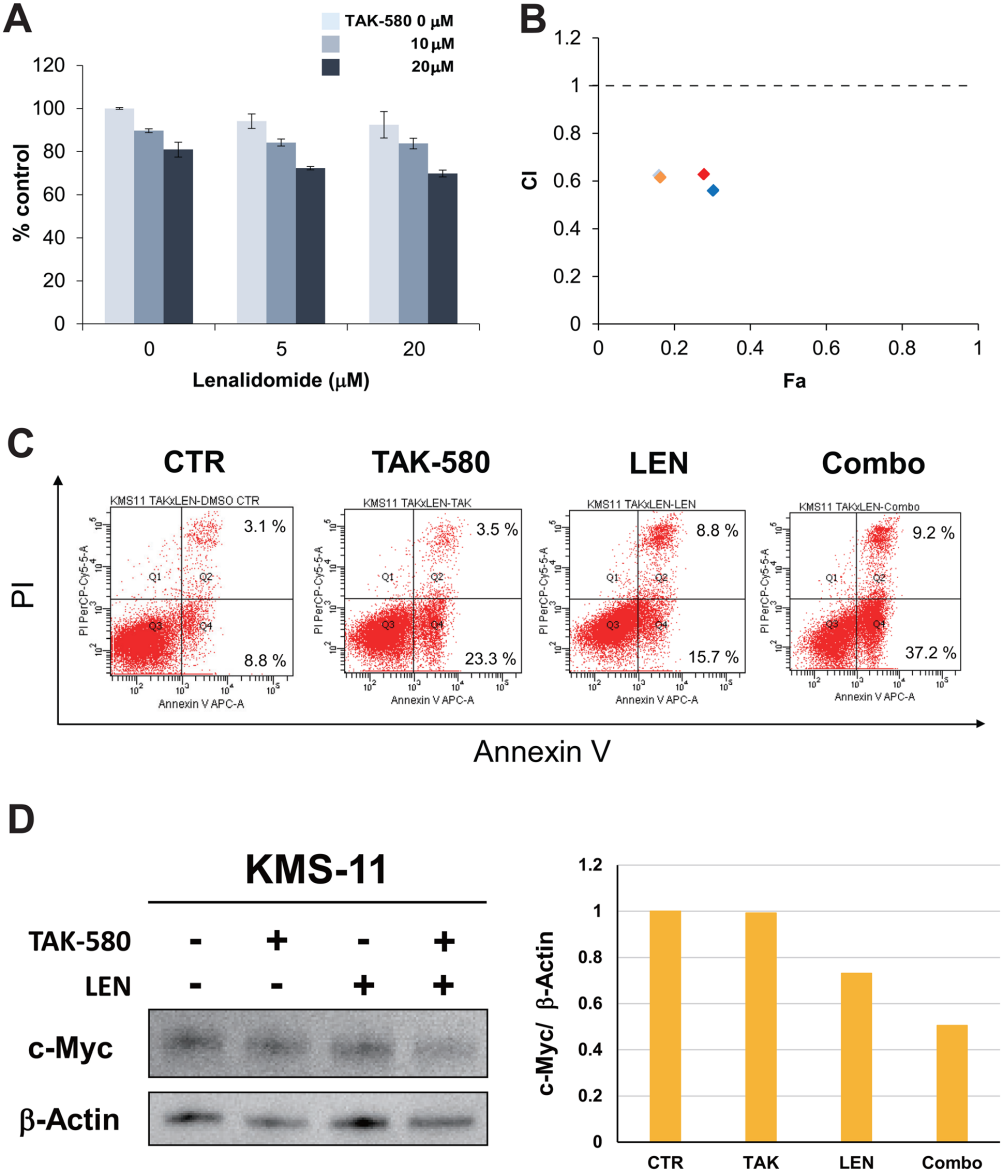
TAK-580 induces synergistic cytotoxicity with lenalidomide (LEN) in MM cells. (**A**) (Left panel): KMS-11 cells were treated with TAK-580 (0–20 μM) in combination with LEN (0–20 μM) for 48 h. In each case, the cell viability of triplicate cultures was assessed with the Cell Titer-Glo^®^ Cell Viability Assay and expressed as the percentage of the untreated control. Data are the mean ± SD. (**B**): Isobologram analysis shows the synergistic or additive cytotoxic effect of TAK-580 and LEN in Figure 5A. Light blue, orange, red, and blue rhombuses indicate CI values of the combination of TAK-580 and LEN; 0.63 (TAK-580 10 μM and LEN 5 μM), 0.62 (TAK-580 10 μM and LEN 20 μM), 0.63 (TAK-580 20 μM and LEN 5 μM), and 0.56 (TAK-580 20 μM and LEN 20 μM), respectively. CI < 1.0 indicates synergism; CI = 1.0 indicates an additive effect; and CI > 1.0 indicates antagonism. CI, combination index; Fa, fraction affected. (**C**) KMS-11 cells were cultured with TAK-580 (20 μM), LEN (40 μM), or TAK-580 plus LEN for 72 h. Apoptotic cells were analyzed with flow cytometry using annexin V/PI staining. Apoptosis was assessed as the percentage of annexin V-positive cells. (**D**) (Left panel): KMS-11 cells were treated with TAK-580 (20 μM) alone or in combination with LEN (20 μM) for 48 h. Whole-cell lysates were subjected to western blotting using c-Myc and β-Actin Abs. (Right panel): The graph represents ratios of c-Myc density relative to β-Actin in Figure 5D.

We next examined the anti-MM effect of TAK-580 in combination with LEN in the KMS-11 MM cell line using the Cell Titer-Glo^®^ Cell Viability assay. The combination of TAK-580 and LEN induced synergistic cytotoxicity in KMS-11 cells (Figure 5A and 5B). In addition, annexin V/PI staining showed that TAK-580 significantly enhanced apoptosis that was induced by LEN in KMS-11 cells (Figure 5C). Moreover, we explored the mechanism of the anti-MM effect of the TAK-580 plus LEN combination. Interestingly, TAK-580 and LEN synergistically inhibited c-Myc, a major cereblon modulated factor and master regulator in MM, in KMS-11 cells (Figure 5D). Taken together, these results indicated that TAK-580 also enhanced LEN-induced MM cytotoxicity and apoptosis in MM cells.

### TAK-580 induces synergistic cytotoxicity with next-generation proteasome inhibitors or immunomodulatory drugs

**Figure 6:**
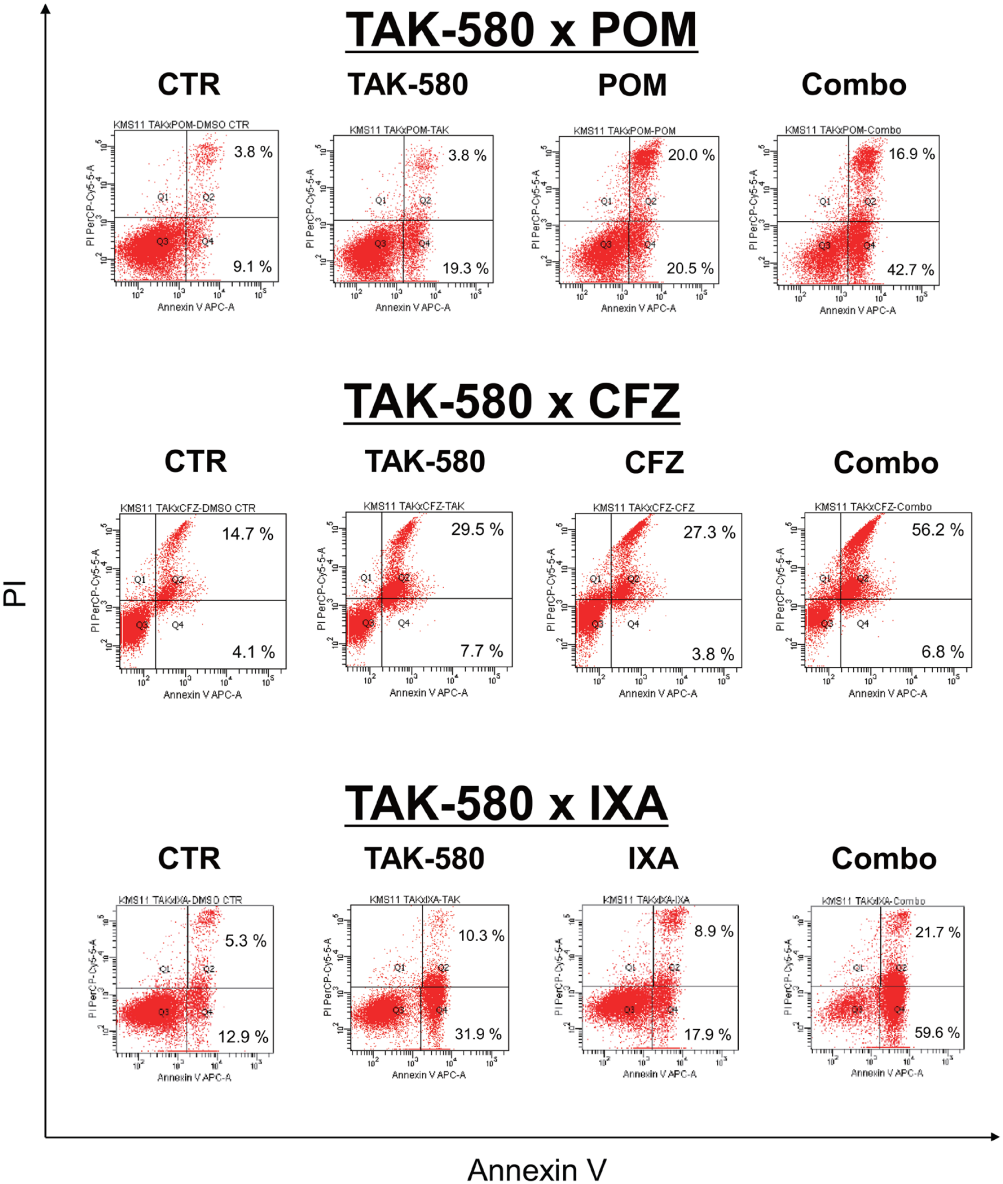
TAK-580 induces synergistic cytotoxicity with other novel agents in MM cells. KMS-11 cells were cultured with TAK-580 (20 μM), POM (40 μM), or TAK-580 plus POM for 72 h; TAK-580 (20 μM), CFZ (1 nM), or TAK-580 plus CFZ for 24 h; TAK-580 (20 μM), IXA (30 nM), or TAK-580 plus IXA for 48 h. Apoptotic cells were analyzed with flow cytometry using annexin V/PI staining. Apoptosis was assessed as the percentage of annexin V-positive cells.

Next, we explored the mechanism of the anti-MM effect of the combination of TAK-580 plus next-generation proteasome inhibitors or immunomodulatory drugs (IMIDs). Interestingly, annexin V/PI staining showed that TAK-580 significantly enhanced apoptosis that was induced by POM, CFZ, or IXA in KMS-11 cells (Figure 6). Taken together, these results indicated that TAK-580 also enhanced the cytotoxicity and apoptosis induced by next-generation proteasome inhibitors or IMIDs in MM cells.

## DISCUSSION

The RAS-RAF-MEK-ERK signaling pathway plays an important role in tumorigenesis, cell proliferation, inhibition of apoptosis, and drug resistance [19, 20]. The *BRAFV600E* mutation contributes to the pathogenesis of malignant melanoma [21]. The combination of a pan-RAF inhibitor and a MEK inhibitor shows an excellent effect on patients with this mutation [21, 22], has already been clinically applied [23], and is considered to be a successful example of inhibiting the RAS-RAF-MEK-ERK pathway. On the other hand, *RAS* mutations may be key players in malignant transformation of clonal plasma cells and myeloma pathogenesis [24]. Importantly, other groups have shown that *NRAS* or *KRAS* mutations confer resistance of various malignancies including MM to typical or molecular targeted chemotherapies [9, 25–27]. Although blocking the RAS-RAF-MEK-ERK pathway was thought to be an attractive option for treating MM [6], the effects of inhibitors targeting the RAS-RAF-MEK-ERK pathway so far have been limited [6, 28], and new-generation RAS inhibitors are awaited. Our group is now focusing on the potential of RAS-RAF-MEK-ERK pathway inhibitors, and we have already reported the synergistic combination effect of the novel selective heat shock protein (HSP)90α/β inhibitor, TAS-116, and RAS-RAF-MEK-ERK inhibitors in *RAS*-mutated or *BRAF*-mutated MM cells [29]. This time, we focused on a novel pan-RAF inhibitor, TAK-580. In the present study, TAK-580 showed a superior anti-myeloma effect compared to the existing drug, dabrafenib, and importantly also showed excellent cytotoxic effects on MM cells that are resistant to multiple regimens. Two classes of RAF inhibitors have been developed: class I *BRAF* mutant-selective inhibitors (vemurafenib, dabrafenib) and class II pan-RAF inhibitors (sorafenib). Importantly, many class I inhibitors bind to the “Asp-Phe-Gly (DFG)-in” active conformation of BRAF, and promote transactivation of wild-type RAF, leading to paradoxical activation of the MAPK pathway and chemoresistance [13, 30, 31]. TAK-580 is a novel class II pan-RAF inhibitor, binds to the inactive, “DFG-out” conformation of BRAF, and potently inhibits both wild-type and mutant RAF kinases, possibly leading to a more effective blockade of the MAPK pathway with minimal paradoxical activation [13, 31]. Therefore, TAK-580 is considered to have the ability to overcome resistance, and is considered to be a very promising drug for the treatment of MM.

FOXO3a is a member of the FOXO subfamily of forkhead transcription factors that mediate a variety of cellular processes including apoptosis, proliferation, cell cycle progression, DNA damage, and tumorigenesis [14]. Importantly, FOXO3a is an important regulator of Bim expression [32], and FOXO3a transcriptional activity is inhibited when phosphorylated [33]. In the present study, we also found that TAK-580 dose dependently inhibited phosphorylation of FOXO3, and induced upregulation of Bim_L_ and Bim_S_ (Figure 3C; Figure 3D). Because BRAF acts upstream of Bim [34], and decreased phosphorylation of FOXO3 leads to FOXO3 activation and subsequent activation of Bim expression, the FOXO3a-Bim axis possibly primes TAK-580-induced apoptosis signaling. Importantly, TAK-580 in combination with BTZ can further enhance apoptotic signaling via the FOXO3a-Bim axis and also the terminal UPR (Figure 4D; Figure 4E; Supplementary Figure 3). These results indicate that TAK-580 alone or in combination with BTZ induces excellent anti-myeloma effects in MM cells. Furthermore, TAK-580 also enhanced cytotoxicity and apoptosis induced by next-generation proteasome inhibitors (CFZ and IXA) or IMIDs (POM) in MM cells (Figure 6). Taken together, TAK-580 is considered to be an excellent chemo-sensitizer for BTZ or next-generation novel agents.

BTZ is still a very important therapeutic component that forms the backbone of MM treatment in real-life practice [35]. Even in daily clinical practice, we often consider possible treatment combinations based on the LEN- and BTZ-sensitive/refractory status [36]. However, the biggest problem is that MM cells eventually become resistant to BTZ via various mechanisms [37]. Although second- and third-generation proteasome inhibitors have been developed, this problem has not been solved. MM is a very heterogeneous disease and includes various subclones. Thus, overcoming the BTZ-resistance mechanism by using this drug in combination with other agents is required [36]. Importantly, *NRAS* mutation significantly reduces myeloma sensitivity to single-agent BTZ therapy [9]. In addition, MM patients harboring oncogenic *KRAS* often have a worse outcome compared with those with *NRAS* mutations or wild-type *RAS* [6, 38]. As one of the mechanisms by which *RAS* mutations induce drug resistance, deregulated RAS-RAF-MEK-ERK activity contributes to anti-apoptotic molecules in MM cells, and is associated with greater relapse, shorter survival, and drug resistance [6, 39]. In the present study, TAK-580 specifically inhibited the RAS-RAF-MEK-ERK pathway and significantly induced anti-myeloma effects and apoptosis in *NRAS*-mutated INA-6 and *KRAS*-mutated RPMI-8226 MM cell lines. These results suggest that TAK-580 partly overcomes BTZ resistance in MM cells.

We also found that TAK-580 showed excellent synergistic anti-MM effects with LEN in MM cell lines via inhibition of c-Myc (Figure 5A–5D). Most importantly, this combination can be administered as an all-oral regimen. MM is a disease of the elderly, and determination of how to treat elderly and fragile patients is a very important point [40]. Novel drugs such as daratumumab and carfilzomib have led to more profound responses and a clear survival prolongation in MM patients [41]. However, aside from regimens that aim for a complete response, protocols aimed at control of symptoms of hypercalcemia, renal failure, anemia, and bone lesions (CRAB) and protocols that emphasize quality of life are also required [42]. The IXA, LEN, and dexamethasone (IRd) regimen is an all-oral, effective triplet regimen [43]. In addition, IRd is also effective for high-risk fluorescence *in situ* hybridization patients in subgroup analysis of the TOURMALINE-MM1 study of IRd versus placebo-Rd in patients with RRMM [44]. Because TAK-580 shows significant anti-myeloma effects in RRMM cells, and LEN has an excellent synergistic effect with various novel agents [45], the combination of TAK-580 and LEN is considered to provide significant synergistic effects, even in aggressive RRMM cases. These results suggest that TAK-580 may also be an excellent chemo-sensitizer for LEN, and the all-oral combination of TAK-580 and LEN enhances anti-myeloma effects even in RRMM cases.

Stratified medicine is becoming more important in the area of MM treatment. The best example is venetoclax for t(11;14) MM [46]. t(11;14), which induces the *IgH*-*CCND1* fusion transcript, is seen in 15–20% of patients with MM, and may be an intermediate risk factor in MM [46, 47]. Venetoclax is a selective, orally bioavailable BCL-2 inhibitor, and has shown excellent effects *in vitro* and *in vivo* for MM cases with t (11;14) [46]. In particular, venetoclax shows significant cytotoxic effects in MM cell lines with *CCND1* translocation, regardless of the mutation status of *TP53* [48]. This example is considered to be a precursor to tailor-made medicine. With the advent of the genome era, advances in genomics and epigenomics are revolutionizing understanding of mechanisms underlying MM with next-generation sequencing and other tests [49, 50]. Although malignant melanoma is the best example, if MM cases that are highly dependent on the RAS-RAF-MEK-ERK pathway can be identified at the first examination, combining BTZ or LEN with a pan-RAF inhibitor in these cases from an early stage is recommended. Whether treatment until the first relapsing phase is successful is a very important point. Clearly, development of further RAS-RAF-MEK-ERK pathway inhibitors is promising for treatment of RAS-RAF-MEK-ERK pathway-dysregulated MM.

In conclusion, TAK-580 alone or in combination with novel drugs showed significant anti-myeloma effects on MM cells *in vitro*, providing the framework for its clinical evaluation to improve the outcome of patients with MM.

## MATERIALS AND METHODS

### Reagents

The pan-RAF inhibitor, TAK-580, was provided by Takeda Pharmaceuticals International Co. (Cambridge, MA, USA). BTZ, LEN, pomalidomide (POM), carfilzomib (CFZ), ixazomib (IXA), and dabrafenib were obtained from Selleck Chemicals (Houston, TX, USA).

### Human cell lines

The human MM cell lines, RPMI-8226 and KMS-11, were purchased from the Japanese Collection of Research Bioresources cell bank (Osaka, Japan). The U266 human cell line was kindly provided by Dr. Shinsuke Iida (Nagoya City University, Aichi, Japan). The interleukin-6-dependent INA-6 human cell line was provided by Dr. Kenneth C. Anderson (Dana-Farber Cancer Institute, Boston, MA, USA). All MM cell lines were cultured in RPMI 1640 containing 10% fetal bovine serum (FUJIFILM Wako, Osaka, Japan), 2 μM l-glutamine, 100 U/mL penicillin, and 100 μg/mL streptomycin (Thermo Fisher Scientific, Waltham, MA, USA), plus 10 ng/mL interleukin-6 only for INA-6 cells.

### Primary cells

MM cells from bone marrow samples from patients, normal B cells from healthy donor peripheral blood, and CD34 cells from cord blood were obtained after informed consent was obtained. This study was performed in accordance with the Declaration of Helsinki and was approved by the Institutional Review Board of the Tokai University School of Medicine. Mononuclear cells were separated using Ficoll-Hypaque density sedimentation, and plasma cells were purified by positive selection with anti-CD138 magnetic-activated cell separation microbeads (Miltenyi Biotec, Bergisch Gladbach, Germany). Normal B cells were purified by positive selection with anti-CD19 and anti-CD20 magnetic-activated cell separation microbeads (Miltenyi Biotec). Cord blood CD34-positive and -negative specimens were primarily prepared using the CD34 Progenitor Cell Isolation Kit (Miltenyi Biotec). CD34-positive cells were then purified again using anti-human CD34 mAbs (Beckman Coulter, Brea, CA, USA), in combination with or without an anti-CD38 antibody (Becton Dickinson, Franklin Lakes, NJ, USA), with a fluorescence activated cell sorting vantage instrument (Becton Dickinson).

### Growth inhibition assay

The growth inhibitory effect of TAK-580 or dabrafenib alone or in combination with novel agents (BTZ or LEN) on MM cell lines, peripheral blood mononuclear cells, and cord blood CD34-positive cells was assessed with Cell Titer-Glo^®^ Cell Viability assays, as indicated by the manufacturer’s protocol.

### Western blotting

MM cells were cultured with or without novel or conventional agents; cells were then harvested, washed, and lysed as reported [51, 52]. Cell lysates were subjected to SDS-PAGE, transferred to membranes, and immunoblotted with the following antibodies: B-Raf, C-Raf, phospho-MEK1/2, MEK1/2, phospho-ERK, ERK, phospho-v-akt murine thymoma viral oncogene homolog (AKT), AKT, cleaved caspase-3, poly (ADP-ribose) polymerase (PARP), phospho-forkhead box O3 (FOXO3), FOXO3, B-cell lymphoma 2 (Bcl-2) interacting mediator of cell death (Bim), cellular myelocytomatosis (c-Myc), CCAAT-enhancer-binding protein homologous protein (CHOP), and β-Actin (all from Cell Signaling, Beverly, MA, USA). Protein expression was quantified using ImageJ (National Institutes of Health, Bethesda, MD, USA).

### Detection of apoptosis with annexin V/propidium iodide (PI) staining

Detection of apoptotic cells was done with fluorescein isothiocyanate- or allophycocyanin-labeled annexin V (BD Biosciences, Franklin Lakes, NJ, USA) and PI (Hoffman-La Roche, Basel, Switzerland), as described [53]. Apoptotic cells were analyzed on a BD FACSCalibur flow cytometer (BD Biosciences). Early apoptotic cells were stained with annexin V but not PI, whereas late apoptotic or necrotic cells were stained with both annexin V and PI.

### Statistical analysis

Statistical significance was determined using the Student’s *t*-test. Probability values of *P* < 0.05 were considered statistically significant. The combination index (CI) values were calculated by isobologram analysis using the CompuSyn Version 1.0 software program (ComboSyn, Paramus, NJ, USA). A CI of less than, equal to, and more than 1.0 indicates synergism, an additive effect, and antagonism, respectively.

## SUPPLEMENTARY MATERIALS


