# Supplementary material for: Anti-tumor activities of the new oral pan-RAF inhibitor, TAK-580, used as monotherapy or in combination with novel agents in multiple myeloma
Source: Oncotarget. 2020 Nov 3;11(44):3984–97. doi: 10.18632/oncotarget.27775 (PMC7646837; doi:10.18632/oncotarget.27775)
Supplement: Supplementary file 1 [file oncotarget-11-3984-s001.pdf]

Anti-tumor activities of the new oral pan-RAF inhibitor, TAK-580, used as monotherapy or in combination with novel agents in multiple myeloma

SUPPLEMENTARY MATERIALS

TAK-580

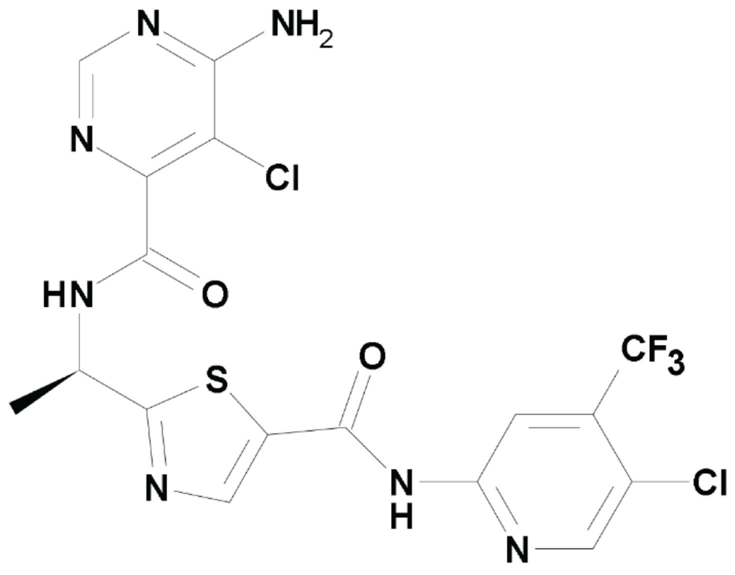

Supplementary Figure 1: Chemical structure of TAK-580.

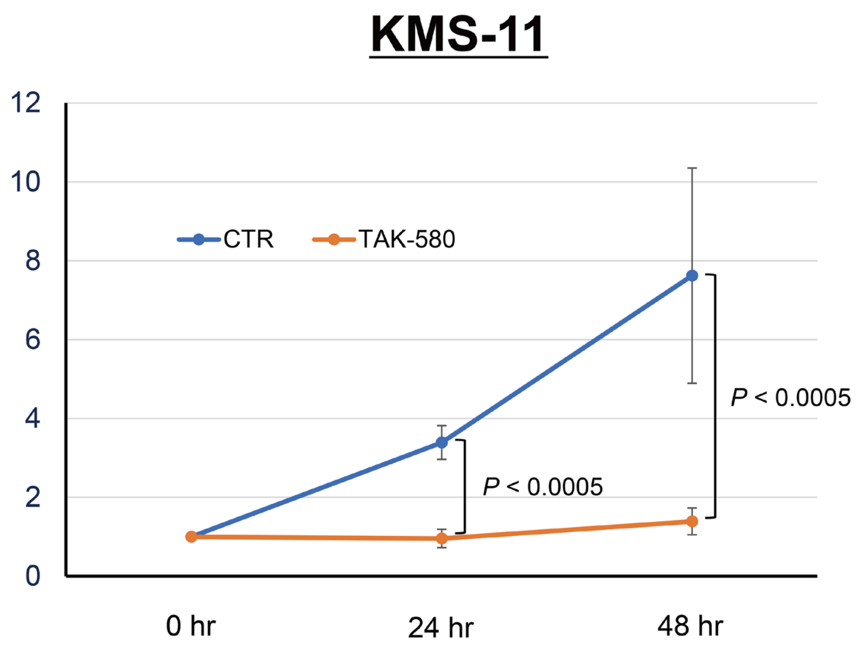

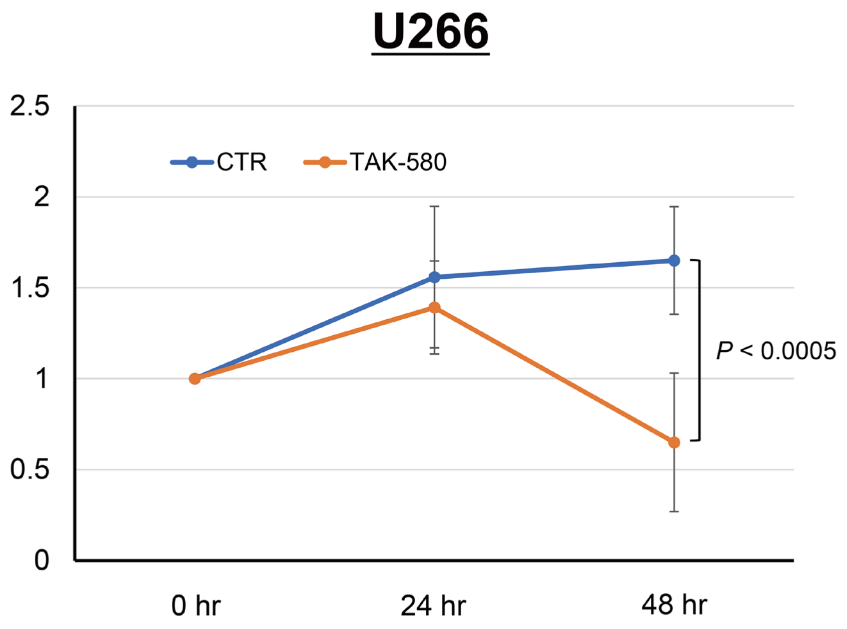

Supplementary Figure 2: Time-dependent effect of TAK-580 in MM cells. KMS-11 and U266 cells were cultured with TAK-580 (20 µM) for 24 or 48 h. The cell number and viability were assessed with trypan blue exclusion. Data are the mean ± SD of six replicate wells.

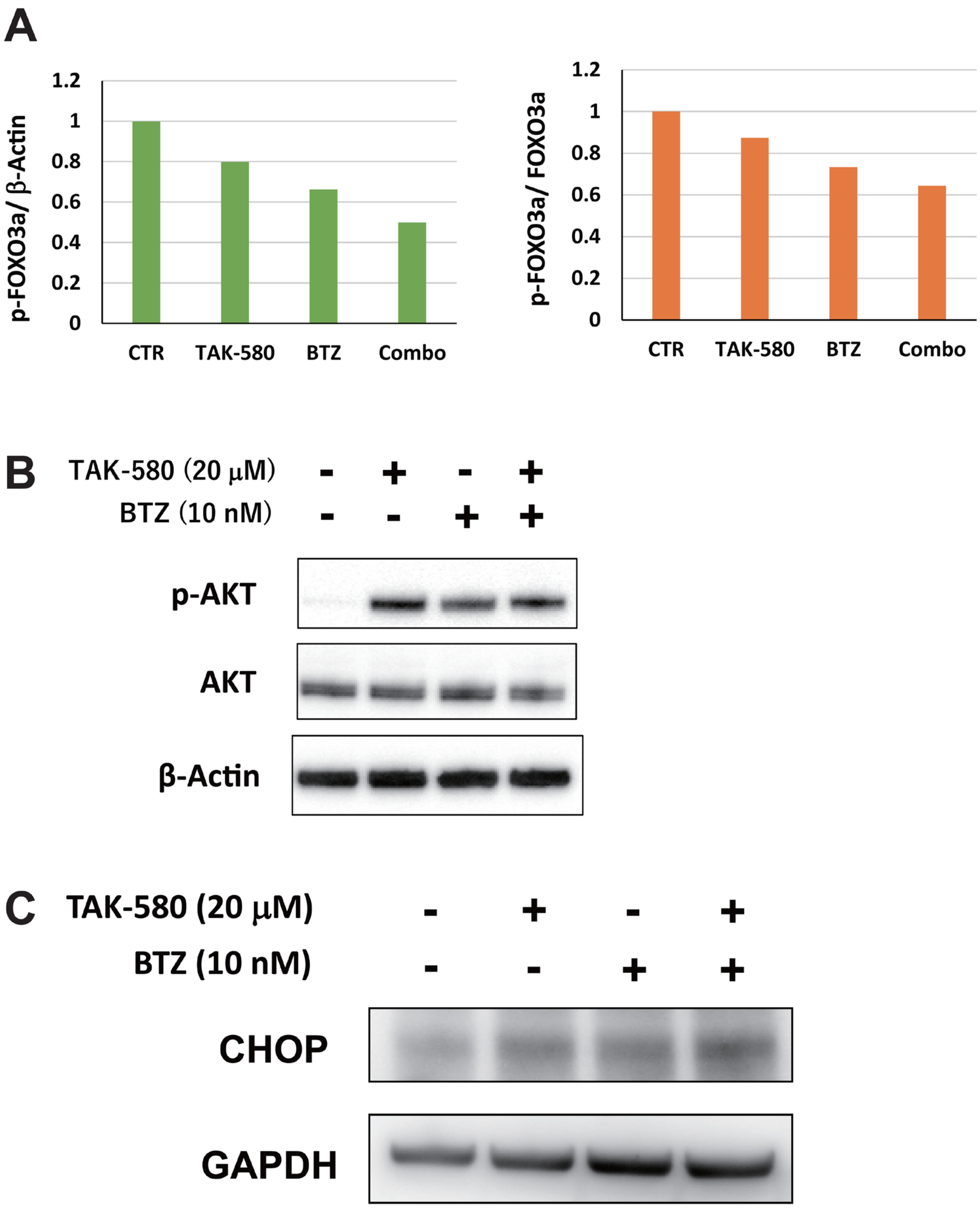

Supplementary Figure 3: The combination of TAK-580 and BTZ triggers synergistic anti-MM activity. (**A**) (Left panel): The graph represents ratios of p-FOXO3 density relative to β-Actin in Figure 4D. (Right panel): The graph represents ratios of p-FOXO3 density relative to FOXO3 in Figure 4D. (**B**) KMS-11 cells were treated with TAK-580 (20 μM) alone or in combination with BTZ (10 nM) for 16 h. Whole-cell lysates were subjected to western blotting using phospho-AKT, AKT, and β-Actin Abs. (**C**) RPMI-8226 cells were treated with TAK-580 (20 μM) alone or in combination with BTZ (10 nM) for 5 h. Whole-cell lysates were subjected to western blotting using CHOP and β-Actin Abs.
